# Malnutrition as a Risk Factor in the Development of Oral Cancer: A Systematic Literature Review and Meta-Analyses

**DOI:** 10.3390/nu16030360

**Published:** 2024-01-26

**Authors:** Romeo Patini, Eleonora Favetti Giaquinto, Gioele Gioco, Raffaella Castagnola, Vittoria Perrotti, Cosimo Rupe, Leonardo Di Gennaro, Giuseppina Nocca, Carlo Lajolo

**Affiliations:** 1 Dipartimento di Testa-Collo ed Organi di Senso, Università Cattolica del Sacro Cuore, 00168 Rome, Italy; romeo.patini@unicatt.it (R.P.); eleonora.favettigiaquinto01@icatt.it (E.F.G.); gioele.gioco@unicatt.it (G.G.);cosimorupe@gmail.com (C.R.); carlo.lajolo@unicatt.it (C.L.); 2 UOC Odontoiatria Generale e Ortodonzia, Dipartimento di Neuroscienze, Organi di Senso e Torace, Fondazione Policlinico Universitario A. Gemelli IRCCS, 00168 Rome, Italy; 3 Department of Medical, Oral and Biotechnological Sciences, “G. d’Annunzio” University of Chieti-Pescara, 66100 Chieti, Italy; v.perrotti@unich.it; 4 Hemorrhagic and Thrombotic Diseases Center, Department of Diagnostic Imaging, Radiotherapy, Oncology and Haematology, Fondazione Policlinico Universitario A. Gemelli IRCCS, 00168 Rome, Italy; leonardo.digennaro@policlinicogemelli.it; 5 Dipartimento di Scienze Biotecnologiche di Base, Cliniche Intensivologiche e Perioperatorie, Università Cattolica del Sacro Cuore, 00168 Rome, Italy; giuseppina.nocca@unicatt.it; 6 Fondazione Policlinico Universitario “A. Gemelli”, IRCCS, Largo Agostino Gemelli 8, 00168 Rome, Italy

**Keywords:** hypovitaminosis, malnutrition, oral cancer, vitamins

## Abstract

This systematic review and meta-analyses aimed to assess whether malnutrition may increase the incidence of oral cancer. Following the PRISMA statement, the research was conducted on PubMed, Scopus, and MEDLINE via OVID without any time restrictions. The risk of bias was assessed, and the quality of evidence for each performed meta-analysis was evaluated using the Grading of Recommendations Assessment, Development and Evaluation (GRADE) framework. Sixty-one articles met the inclusion criteria and seven studies underwent quantitative evaluation. For our meta-analysis on hypovitaminosis B, three studies with a total of 90,011 patients were included. An odds ratio of 2.22 was found. Our meta-analysis on the correlation between vitamin C and oral cancer included one study with a total of 866 patients and the derived odds ratio was 1.06. Our meta-analysis on the relationship between vitamin D deficiency and the incidence of oral cancer included three studies with a total of 12,087 patients and the odds ratio was −2.58. The GRADE system showed a moderate strength of evidence due to the presence of studies with a high risk of bias and high indirectness of the data given. The present findings suggest that an inadequate intake of vitamins, particularly vitamin D, poses a risk for the onset of oral cancer.

## 1. Introduction

According to the official data from the World Health Organization (WHO), in 2020, 377.713 new cases of oral and lip carcinoma were diagnosed, making it the sixteenth most common cancer worldwide [1]. Despite advancements in precision diagnostic tools and increasingly effective therapeutic strategies, oral and lip carcinomas still carry a poor prognosis. Approximately 50% of individuals die within five years, while the remaining 50% experience aesthetic and functional impairments that significantly impact their quality of life [2]. Historically, the main risk factors for this type of cancer include the male gender, bad habits such as smoking, alcohol consumption, and betel nut use, potentially malignant disorders, immunodeficiency, as well as hypovitaminosis and nutritional deficiencies [3].

Malnutrition, according to the WHO, is a condition of imbalance between the intake of energy and nutrients and the body’s specific needs for growth and for the maintenance of its functions. The term malnutrition is generally used as a synonym for “undernutrition”, but strictly speaking, malnutrition (which literally means “poor nutrition”) can also refer to overnutrition, obesity, and imbalances in nutrient intake or their toxicity [4]. According to the WHO, undernutrition or malnutrition is the most significant global health threat, upon which many other local diseases depend.

The primary cause of malnutrition is the nutritional inadequacy of available food, but it can also result from concurrent illnesses that alter the body’s ability to absorb nutrients, leading to a reduced nutritional intake. While abnormalities in the body’s protein content (lean tissues) and energy (mainly fats) and discrepancies in the supply and demand of proteins and/or energy are often important characteristics of various forms of undernutrition, these alone may not capture the entire spectrum of malnutrition [5]. This spectrum includes specific deficiencies of nutrients (specific nutrient malnutrition), combinations of nutrients (such as vitamins, minerals, proteins, or trace elements), or an imbalance between nutrients (e.g., fats, carbohydrates, proteins), all of which can potentially lead to negative clinical and functional effects on the human body.

Based on the causes, malnutrition can be classified into:

1. Primary malnutrition, linked to the availability and intake of food;
2. Secondary malnutrition, resulting from specific physiological (e.g., pregnancy, lactation, febrile states) and pathological conditions that impair digestion and nutrient absorption [6].

Another classification of malnutrition considers the types of nutrients that the body is deficient in. Depending on the inadequate intake of one or more elements such as calories, proteins, vitamins, or minerals, we can talk about protein-energy malnutrition, vitamin malnutrition, or salt malnutrition. The most widespread form of primary global undernutrition is protein–energy or protein–caloric malnutrition. It occurs worldwide in the pediatric or geriatric population that lacks access to nutritious substances. It encompasses two pathological forms: kwashiorkor and marasmus. Kwashiorkor is a condition caused by a diet that is energetically sufficient but deficient in essential amino acids, vitamins, and minerals, while marasmus is caused by both energy and protein deficiency [7]. Salt malnutrition is caused by a lack of essential micronutrients, namely minerals, due to reduced absorption, particularly of iron and calcium [8], or to a reduced quantity of micronutrients, such as iodine and selenium, in a specific geographical area [9]. Vitamin malnutrition is caused by a prolonged deficit in the body’s intake. In particular, partial vitamin deficiencies are referred to as hypovitaminoses, while total deficiencies are referred to as avitaminoses.

In the scientific literature, malnutrition is often reported as a risk factor for the onset of oral cancer [1,3]. In this regard, exposure to the condition of hypovitaminosis appears to increase cancer incidence, including squamous-cell carcinomas of the oral mucosa. Studies have shown that a deficiency in vitamins and other nutrients can negatively impact the immune system and DNA repair mechanisms, making cells more susceptible to tumor transformations [1,4]. The importance of proper nutrition and adequate intake of vitamins is recognized in helping to prevent the development of diseases, including cancer. Therefore, paying attention to the diet and nutritional balance is essential to reduce the risk of developing oncological conditions.

The aim of this study is to assess, through a systematic literature review and meta-analyses, whether hyponutritional states resulting in micronutrient deficiencies should be considered risk factors for carcinogenesis in the head and neck region.

## 2. Materials and Methods

In this systematic review, the adopted protocol followed the Preferred Reporting Items for Systematic Reviews and Meta-Analyses (PRISMA) guidelines. This review is registered on PROSPERO with code CRD42023466459.

### 2.1. Study Objective and PECOS Model

The objective of this study was defined in accordance with the PECOS format. This model is used by the scientific community to formulate the objectives of scientific studies in an organized and repeatable manner. The method is based on five elements to which the acronym refers:

- P. Population/problem/patient: adult patients not affected by any systemic disease;
- E. Exposure: patients exposed to primary or secondary malnutrition due to different causes;
- C. Comparison: the incidence of oral cancer in healthy subjects and in patients not affected by primary or secondary malnutrition;
- O. Outcome: development of oral carcinoma;
- S. Study design: cohort studies, case–control studies, cross-sectional studies, and randomized clinical trials (RCTs) with no fewer than 10 patients.

#### 2.1.1. Inclusion Criteria

The search was limited to studies published in English without any restriction regarding the year of publication.

#### 2.1.2. Exclusion Criteria

The following exclusion criteria were applied: studies with a population of fewer than 10 patients, in vivo or in vitro animal model studies, systematic reviews, and articles published in languages other than English, Italian, French, and Spanish.

The objective that this systematic review aimed to address was to answer the following question: “Do patients affected by malnutrition have an increased risk of developing squamous cell carcinoma of the oral mucosa?”.

##### Electronic Search

The research was conducted on 3 databases (PubMed, Scopus, and MEDLINE via OVID) without any time restrictions up to November 2023 using a combination of keywords and MeSH terms, as follows: [“oral cancer” AND “malnutrition” AND “hypovitaminosis”]; [(“oral carcinoma” OR “oral cancer” OR “oral neoplasm”) AND folate]; [(“oral carcinoma” OR “oral cancer” OR “oral neoplasm”) AND (“folic acid”)]; [(malnutrition OR hypovitaminosis OR vitamin deficiency OR Protein-Energy Malnutrition OR metabolic disease OR nutritional disorder) AND “Oral Cancer”]. The last search was conducted on 11 November 2023. The reported search strategy was used for PubMed and then adapted for Scopus and MEDLINE via OVID.

##### Manual Search

In addition to the electronic search, a hand search was undertaken by checking the references of the included studies to identify further eligible studies. In addition, a manual search was also conducted in the journals *Oral Oncology*, *Oral Diseases*, *Lancet Oncology*, *Journal of Oncology, Oral Pathology*, and *Nutritional Cancer* for articles published from the journals’ inceptions up to 2023.

##### Unpublished Articles

The unpublished literature was searched in the clinical study registry of the National Institutes of Health in the United States and the European multidisciplinary database to identify historical studies and grey literature. The bibliographic references of all included articles were also checked in a similar manner to identify additional potentially relevant studies and increase the sensitivity of the search.

### 2.2. Study Selection

Based on the inclusion criteria, the recovered citations were independently reviewed by two authors (E.F.G. and R.P.), and relevant studies were identified based on title and abstract. If those did not provide sufficient information about the inclusion criteria, the full text was evaluated to assess eligibility. Disagreements were solved through discussion, and a third senior author (C.L.) was consulted to make final decisions.

To ascertain the reviewers’ level of agreement, the Cohen’s kappa coefficient was calculated [10]. The level of agreement is considered excellent when k is greater than 0.75, fair-to-good when it falls between 0.40 and 0.74, and poor when it is less than 0.4.

All articles that met the inclusion criteria were subjected to data extraction and quality assessment. All irrelevant articles were excluded, and the reasons for exclusion were described for the articles that were read in their entirety.

#### 2.2.1. Data Extraction

Data were collected using a specifically constructed data extraction form. In cases where the publication did not provide all the necessary data, the corresponding author was contacted via email to obtain the missing information.

In the case of redundant publications, the most recent article or the one with the highest number of enrolled patients was included.

#### 2.2.2. Quality Assessment

The risk of methodological errors (bias) in the included studies was assessed independently and in duplicate by two authors (R.P and E.F.G.) as part of the data extraction process.

The evaluation of bias risk was performed using the Newcastle–Ottawa Scale (NOS) [11] for case–control, cross-sectional, and cohort studies, whereas the Cochrane Collaboration Tool [12] was used for evaluating randomized controlled trials. For the NOS, the presence of each parameter was recorded with a green circle with a “+”, while its absence was recorded with a red circle with a “−”. Articles with 1–3 stars were classified as having a high risk of bias, those with 4–6 stars as having a medium risk, and those with 7–8 stars as having a low risk. An additional analysis of the overall quality of evidence for each meta-analysis was independently conducted by the same examiners using the Grading of Recommendations, Assessment, Development, and Evaluations (GRADE) system. Any discrepancies between the two reviewers (E.F.G. and R.P.) were resolved through discussion with a supervising author (C.L.). In the case of meta-analyses with fixed-effect model, publication bias was assessed through a funnel plot, created using Review Manager (Revman), Version 5.4 [13] software.

#### 2.2.3. Heterogeneity Assessment

Review Manager 5 software was used to assess the heterogeneity of the included studies in each conducted meta-analysis [13]. The authors calculated the comparability of the proportions observed among the results using only the case with the I2 test. If the *p*-value was less than 0.1, heterogeneity was considered significant. Additionally, the same test was used as a measure of heterogeneity among the studies with the following scheme:

- 0−40%: negligible;
- 30−60%: moderate;
- 50−90%: substantial;
- 75−100%: considerable [14].

### 2.3. Data Analysis

Meta-analyses were conducted only when homogeneous studies that reported results consistently and comparably were found. In such cases, meta-analyses were performed using a fixed-effects model. Only in the presence of substantial heterogeneity among the included studies (>50%) was a random-effects model used. A forest plot was created to depict the effects of individual studies on the meta-analyses and the overall estimates. Review Manager 5 [13] was used to perform all the analyses. The significance threshold was set at *p* < 0.05.

## 3. Results

### 3.1. Study Selection

**Figure 1:**
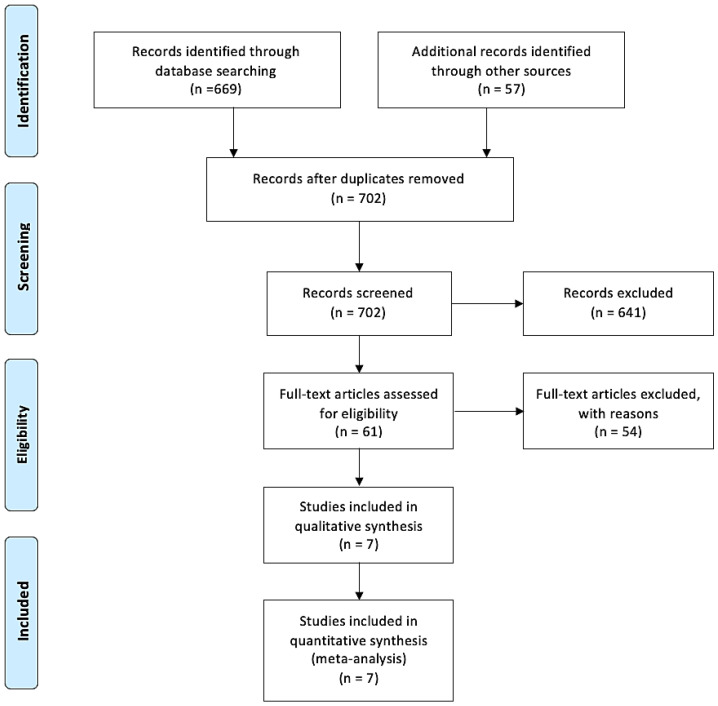
PRISMA flow chart from [15].

Figure 1 shows the flowchart of the research and study selection process.

A total of 726 articles were identified, with 669 found through electronic searches and 57 from other sources. After removing duplicates, 702 studies remained for selection. Out of the 702 articles analyzed, 641 were excluded after reviewing the titles and abstracts. Eventually, out of the remaining sixty-one articles selected for full-text evaluation, seven met the inclusion criteria and were included in the qualitative and quantitative analyses (meta-analyses).

### 3.2. Study Characteristics

The characteristics of the studies included in our meta-analyses are summarized in Table 1.

### 3.3. Risk of Bias Assessment

**Figure 2:**
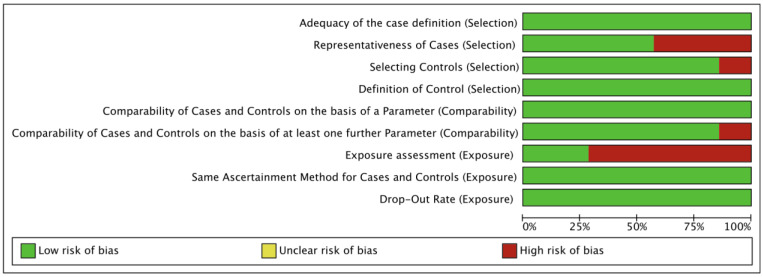
Risk of bias summary.

**Figure 3:**
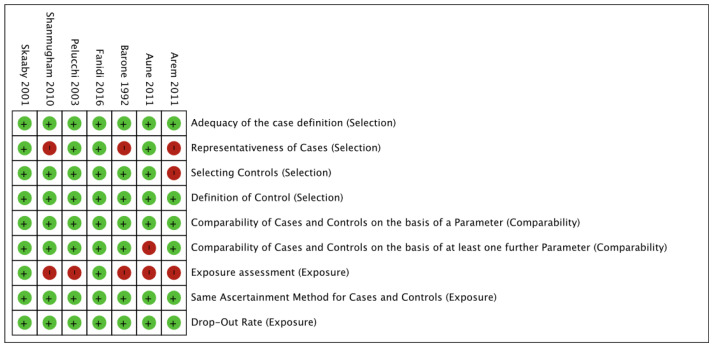
Risk of bias graph [16,17,18,19,20,21,22].

The risk of bias assessment is summarized in Figure 2 and Figure 3. Methodological quality was high for five studies and moderate for two studies.

The GRADE system provides information on the certainty of conclusions and the strength of evidence and is reported in Table 2.

### 3.4. Results of the Meta-Analyses

**Figure 4:**
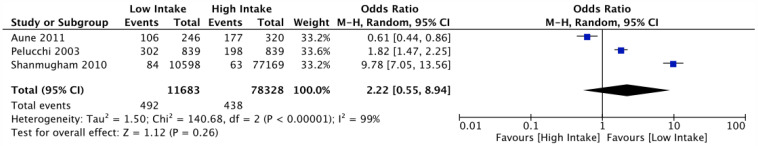
Results of the meta-analysis on the relationship between oral cancer and vitamin B [18,19,21].

Three separate meta-analyses were conducted. The first one (Figure 4) reports data concerning studies on the correlation between folic acid and oral carcinoma. Three studies with a total of 90,011 patients were included; an odds ratio of 2.22 (C.I: 0.55–8.94) was found.

**Figure 5:**
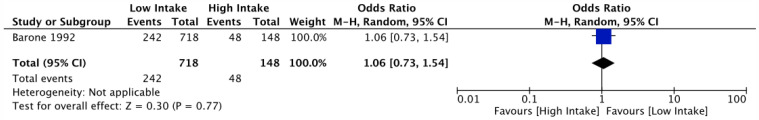
Results of the meta-analysis on the relationship between oral cancer and vitamin C [16].

The second meta-analysis focused on the correlation between vitamin C and oral cancer and included one study with a total of 866 patients (Figure 5). The derived odds ratio was 1.06, with a confidence interval between 0.73 and 1.54.

**Figure 6:**
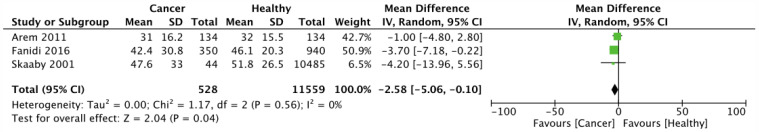
Meta-analysis on the correlation between oral cancer and vitamin D [17,20,22].

The third meta-analysis concerned the relationship between vitamin D deficiency and the incidence of oral cancer. It included three studies with a total of 12,087 patients (Figure 6). The odds ratio was −2.58, with a confidence interval between −5.06 and −0.10.

## 4. Discussion

The relationship between malnutrition and oral cancer carcinogenesis has been investigated in several studies over the years [23,24,25,26]. The present systematic review has highlighted how various forms of vitamin deficiencies leading to frank hypovitaminosis can have different impacts on the risk of developing oral cancer. The data suggest distinct relationships between hypovitaminosis B, C, and D and the risk of oral carcinoma development. These findings particularly underscore the potential for implementing primary prevention screening in the population to identify individuals with nutritional deficiencies and intervene at an early stage of neoplastic risk. Population screening protocols would allow for the identification of individuals at higher risk of developing squamous-cell carcinomas of the oral mucosa by evaluating specific blood parameters and providing vitamin supplementation to further reduce the risk of neoplasms. In particular, the statistical analysis and meta-analysis revealed a statistically significant correlation between vitamin D deficiency and an increased risk of cancer. It is important to underline that the overall vitamin D status of a patient depends on the conversion of cholecalciferol and ergocalciferol to 25-hydroxyvitamin D at the skin level following exposure to ultraviolet radiation [27]. Therefore, it is not solely the nutritional component that determines the vitamin’s blood concentrations [28]. Nonetheless, the administration of vitamin supplements and the consumption of foods rich in calcitriol allow for maintaining higher blood levels above the cutoff of 20 ng/mL in conditions of limited sun exposure, thereby ensuring a proper plasma concentration of the vitamin [14].

Our meta-analysis considered three studies with a total of 12,087 patients to evaluate whether vitamin D deficiency could be considered a risk factor for carcinogenesis. The results showed statistical significance in the data analysis, leading to the conclusion that plasma levels below 20 ng/mL may increase the risk of squamous-cell carcinomas in the oral cavity. In this regard, these results align with existing studies in the literature, which report that vitamin D deficiency is a risk factor for the development of oncological conditions [23,29]. Specifically, this study highlights that squamous-cell carcinoma is one of the specific tumors susceptible to vitamin D blood concentrations and emphasizes how the nutritional component also plays a role in determining serum levels of vitamin D. Thus, the diet also holds significance in maintaining adequate systemic levels of the vitamin [30].

The results of our meta-analyses on the role of vitamin C and vitamin B deficiencies are different. The findings lack statistical significance, preventing the assertion that frank B or C hypovitaminosis is a risk factor for oral cavity carcinoma. When compared with the existing literature, these results indicate that it is not a specific link between oral cavity carcinoma and B or C hypovitaminosis but rather an association with the systemic risk of developing oncological diseases [5,23].

Moreover, the evaluation of at-risk patients would require, similarly to vitamin D, an analysis of plasma concentrations of the vitamins rather than relying on dietary questionnaires. In fact, establishing a plasmatic concentration cutoff value below which a patient could be considered at higher risk of developing neoplastic conditions was not performed in the articles dealing with vitamin B and C deficiencies. Therefore, FFQs (Food Frequency Questionnaires) might only be useful in an initial screening phase, but further biochemical investigations would be necessary to assess precise measurements of folate and ascorbic acid levels in patients for a more accurate identification of at-risk individuals.

Some methodological considerations have emerged from this systematic review. Firstly, it is essential to emphasize that the condition of “malnutrition” was based only on vitamin deficiency instead of its extensive meaning (overnutrition, obesity, and imbalances in nutrient intake or their toxicity) [4]. Consequently, our meta-analyses focused specifically on the correlation between vitamin malnutrition and the increased risk of squamous-cell carcinoma of the oral mucosa.

From a methodological perspective, the studies included in our meta-analysis on vitamin D were considered of high quality, as they did not present data collection biases since the analysis of vitamin D values was directly performed on blood samples from patients. On the other hand, the situation was different for meta-analyses analyzing data on vitamin C and B, where preliminary data were retroactively based on FFQs administered to patients. This might explain the reason why the abovementioned meta-analyses did not show a statistically significant relationship between vitamin C and B deficiencies and oral cancer risk but rather an association with systemic conditions of increased general cancer risk.

The limitations of this study are attributed to both the data collection methods used in the various studies involved and their methodological quality. In fact, for our meta-analyses on vitamins C and B, only articles published between 1992 and 2010 where aggregated data were presented, requiring their reprocessing to perform the meta-analysis, were utilized. This further limitation deeply reduced the reliability of our analysis.

It is also important to underline that the authors carried out a preliminary selection of which micronutrients to consider in the present systematic review and meta-analyses. In fact, there are other vitamins and micronutrients that might be associated with the risk of oral carcinogenesis, such as vitamin A, which is essential for normal cellular growth and differentiation. Laboratory data have demonstrated the potent antiproliferative and differentiation-inducing effects of vitamin A and its synthetic analogues (retinoids) [31]. Different articles report how they may have a prophylactic and a therapeutic role in cancers of epithelial origin [32]. In addition, some studies in the literature report that vitamin A and its derivatives can be used for prophylactic and therapeutic purposes in precancerous conditions [33]. The decision not to include the association between precancerous lesions and vitamin deficiency was made by the authors because the primary objective of this study was to assess only oral carcinoma in relation to vitamin deficits.

The authors therefore also analyzed, in the first instance, the correlation between vitamin A hypovitaminosis and the risk of squamous-cell carcinoma of the oral cavity. They assessed the quality of the studies found. For the assessment of the articles, their materials and methods were analyzed, with particular attention given to the data extraction procedures presented. Articles were excluded from our study if it was not possible to identify a specific blood parameter reference cutoff for evaluating the condition of hypovitaminosis. For this reason, articles in which the sole parameter for assessing vitamin deficiency was based on food questionnaires were not considered. The consequence of this preliminary research led to the exclusion of this specific micronutrient from the systematic review and meta-analysis: the aforementioned articles typically reported an analysis based exclusively on dietary questionnaires [34,35,36,37,38], which, lacking a serum analysis of the micronutrient, did not allow the evaluation of a reference cutoff value below which vitamin A deficiency could be considered a risk factor.

It was observed that in some articles, vitamin A was not presented as an independent risk factor [39] but in association with other known risk factors such as smoking and alcohol consumption, thus preventing the possibility to analyze the sole impact of its deficiency on the evaluated outcome. Indeed, vitamin A is often associated with cigarette smoking as an oncogenic risk factor. It is interesting, moreover, to assess how in some studies, vitamin A and its retinoid derivatives, along with beta-carotene, are considered risk factors for an increased incidence of tumorous pathologies, particularly those related to the lungs [40].

Therefore, vitamin A deficiency was not included in this systematic review and meta-analyses.

The correlation between vitamin deficiency and oral squamous-cell carcinoma is a topic of growing interest in the scientific community. A number of studies have highlighted a relationship between low levels of vitamins, especially vitamin D, and an increased risk of developing oral cancer [14,24,26,41]. This correlation may be attributed to the role of vitamin D in immune system regulation and inflammation prevention, which are key factors in the oral carcinogenesis process. Further research is needed to support and delve deeper into this relationship, but these initial studies suggest that an adequate vitamin intake may play a significant role in preventing oral squamous-cell carcinoma.

However, our research has highlighted the need for further investigations regarding the relationship between other forms of vitamin deficiency, especially vitamin B and C, and oral carcinogenesis. The scarcity of sources results in the inability to establish scientific evidence, which does not allow us to associate the risk of oral cancer with severe vitamin deficiency. As a result, in studies on special oral and maxillofacial pathology, it would be appropriate to emphasize vitamin D deficiency, rather than general vitamin deficiency, as a risk factor. Given that vitamin D is not solely related to nutrition, it cannot be asserted that malnutrition constitutes a risk factor for oral cancer.

## 5. Conclusions

The results obtained from this systematic review and the meta-analyses of the articles included in this study indicate that vitamin malnutrition, specifically vitamin D deficiency, is a risk factor for the development of oral cancer. Therefore, it is crucial to focus on patients with frank hypovitaminosis in order to identify deficiency situations and prevent the onset of neoplastic conditions.

Further studies are needed to better investigate this relationship and explore whether other malnutrition conditions may also be considered as risk factors for the occurrence of squamous-cell carcinoma of the oral cavity.

## Figures and Tables

**Table 1:** Characteristics of articles included in meta-analyses [16,17,18,19,20,21,22]. P = Prospective, R = Retrospective.

Authors–Year	Place of Publication	Study Design	Number of Patients	Analyzed Micronutrient	Patients with SCC	Study Objective	Years of Follow-Up	Results
Barone (1992) [16]	USA	R	870	Vitamin C and E	290	Use of vitamin supplements and the risk of oral and esophageal carcinogenesis	0	It was noted that the intake of vitamin C and E acts as a protective factor in the onset of oral and esophageal cancer.
Skaaby (2001) [17]	Denmark	P	12,204	Vitamin D	1248	Correlation between blood vitamin D concentration and generalized risk of carcinogenesis	11.3	There was no observed increase in the risk of a specific tumor, but a generalized risk of the development of neoplasms.
Pelucchi (2003) [18]	Italy and French Switzerland	R	2521	Folic acid	749	To assess whether daily intake of folic acid is considered a protective factor for SCC	5	Low intake of folic acid and alcohol consumption increase the risk of SCC.
Shanmugham (2010) [19]	USA	P	87,621	Folic acid	147	Quantifying folate intake as a risk factor for the development of SCC	26	Patients who consume >30 g/day of alcohol and <350 μg/day of folate have a 20% increased risk of developing SCC.
Arem (2011) [20]	Finland	P	29,133	Vitamin D	348	Assessing serum vitamin D concentrations as a risk factor for the development of HN tumors	20	No correlation was found between low serum levels and an increased incidence of HNC.
Aune (2011) [21]	Uruguay	R	5571	Folic acid	3539	Folic acid as a protective factor against 11 types of cancer	8	There is a significant reduction in the incidence of oral and pharyngeal SCC in patients with adequate folate intake.
Fanidi (2016) [22]	Europe	P	385,747	Vitamin D	497	Correlation between circulating vitamin D and the incidence and survival of SCC	8	Reduced levels of circulating vitamin D are correlated with an increase in the incidence of laryngeal, hypopharyngeal, and oral cancer.

**Table 2:** GRADE on data included in meta-analyses on the relationship between oral cancer and malnutrition.

Quality Assessment Outcome: Oral Cancer Incidence in Patients with Malnutrition						
Question: Does malnutrition have an influence on oral cancer incidence?						
Number of studies according to meta-analysis	Study design	Risk of bias	Inconsistency	Indirectness	Imprecision	Publication bias
Meta-analysis on folate effects (3 studies)	cohort and case–control studies	medium	serious (a)	serious (b)	not serious	N/A
Meta-analysis on vitamin C effects (1 study)	case–control study	medium	N/A	serious (b)	not serious	N/A
Meta-analysis on vitamin D effects (3 studies)	cohort and case–control studies	medium	not serious	serious (b)	not serious	N/A

(a) Due to high heterogeneity across studies. (b) Due to data indirectness.

## Data Availability

The data presented in this study are available in this article.
